# Hyperacusis: Focus on Gender Differences: A Systematic Review

**DOI:** 10.3390/life13102092

**Published:** 2023-10-21

**Authors:** Lucia Belen Musumano, Stavros Hatzopoulos, Virginia Fancello, Chiara Bianchini, Tiziana Bellini, Stefano Pelucchi, Piotr Henryk Skarżyński, Magdalena B. Skarżyńska, Andrea Ciorba

**Affiliations:** 1 ENT and Audiology Unit, Department of Neurosciences and Rehabilitation, University of Ferrara, 44121 Ferrara, Italy; lbmusumano@gmail.com (L.B.M.); sdh1@unife.it (S.H.); chiara.bianchini@unife.it (C.B.); stefano.pelucchi@unife.it (S.P.); andrea.ciorba@unife.it (A.C.); 2 Centre for Studies on Gender Medicine, University of Ferrara, 44121 Ferrara, Italy; tiziana.bellini@unife.it; 3 Institute of Sensory Organs, 1 Mokra Street, 05-830 Kajetany, Poland; piotrhskarzyski@gmail.com (P.H.S.); m.skarzynska@csim.pl (M.B.S.); 4 Department of Teleaudiology and Screening, World Hearing Center, Institute of Physiology and Pathology of Hearing, 10 Mochnackiego Street, 02-042 Warsaw, Poland; 5 Heart Failure and Cardiac Rehabilitation Department, Faculty of Dental Medicine, Medical University of Warsaw, 8 Kondratowicza Street, 03-242 Warsaw, Poland; 6 Center of Hearing and Speech Medincus, 05-830 Warsaw, Poland; 7 Pharmacy Department, Department of Pharmacotherapy and Pharmaceutical Care, Medical University of Warsaw, 02-042 Warsaw, Poland

**Keywords:** hyperacusis, tinnitus, gender

## Abstract

Background: While gender differences of several diseases have been already described in the literature, studies in the area of hyperacusis are still scant. Despite the fact that hyperacusis is a condition that severely affects the patient’s quality of life, it is not well investigated; a comprehensive understanding of its features, eventually including gender differences, could be a valuable asset in developing clinical intervention strategies. Aim: To evaluate gender differences among subjects affected by hyperacusis. Methods: A literature search was conducted focused on adult patients presenting hyperacusis, using the MedLine bibliographic database. Relevant peer-reviewed studies, published in the last 20 years, were sought. A total of 259 papers have been identified, but only 4 met the inclusion criteria. The review was performed according to the Preferred Reporting Items for Systematic Reviews and Meta-Analysis (PRISMA) guidelines. Results: The four selected papers included data from 604 patients; of these, 282 subjects resulted as affected by hyperacusis (125 females and 157 males). Questionnaires for analyzing factors affecting the attentional, social and emotional variance of hyperacusis (such as VAS, THI, TSCH, MASH) were administered to all included subjects. The data suggest that there are no hyperacusis gender-specific differences in the assessed population samples. Conclusions: The literature data suggest that males and females exhibit a similar level of hyperacusis. However, in light of the subjective nature of this condition, the eventual set up of further tests to assess hyperacusis features could be very helpful in the near future.

## 1. Introduction

Hyperacusis, also referred as noise sensitivity or sound sensitivity, is an abnormally disproportionate increase in loudness perception in response to auditory stimuli of normal volume.

Hyperacusis has several potential features, which are not mutually exclusive, and patients are likely to be heterogeneous. The hyperacusis prevalence is reported to range between 9–15% [1,2,3]. So far, the lack of robust epidemiological data has generated a major information gap among the published literature on this topic.

To focus on this issue, it is necessary to differentiate hyperacusis from another perceptional manifestation, namely recruitment, characterized by the development of abnormal response with the sound level, which becomes progressively louder with rising of intensity, and expression of cochlear disfunction [4]. On the other end, the mechanism underlying hyperacusis is mainly unknown and represents the expression of maladaptation of the central auditory pathway to sounds, in which the limbic system may play a critical role [4]. 

In addition, hyperacusis needs to be distinguished from phonophobia and misophonia, which express the intolerance to different specific ranges of sounds with emotional associations.

Moreover, hyperacusis is a condition that can be associated with tinnitus, acoustic trauma, migraine, facial paralysis, drug-interaction (especially benzodiazepines), and many more. 

Moreover, quantifying the specific discomfort associated with hyperacusis can be challenging, especially when it manifests within a more complex clinical context, such as tinnitus. Several instruments, such as specific self-assessment questionnaires, have been developed to provide a subjective evaluation.

The Tinnitus Handicap Inventory Test (THI) is performed used to analyze the factors affecting the attentional, social, and emotional aspect of life in patients complaining of tinnitus, and it is often administrated in patients affected by hyperacusis as well [5]. Other tools are the Multiple-Activity Scale for Hyperacusis (MASH) [6] and the Visual Analogue Scale (VAS) [7], Hyperacusis Questionnaire [8], Inventory of Hyperacusis Symptoms [9], Hyperacusis Handicap Questionnaire [10], Short Hyperacusis Questionnaire [11], and Hyperacusis Impact Questionnaire [12].

In addition, uncomfortable loudness levels (ULLs) or loudness discomfort levels, are valuable to assess this condition and its severity by evaluating which sounds are perceived to be “bothersome” [13]. The ULLs appear to be correlated with the abnormal sound perception: the average ULL value across the audiometric frequencies is about 100 dB HL in people with normal hearing [14], while lower ULL values in one or both ears are common findings in patients with hyperacusis [15].

At present, the role of gender in hearing hypersensitivity has not been investigated; a comprehensive understanding of all hyperacusis features eventually including gender differences could be important in clinical practice as a valuable asset in developing clinical intervention strategies. 

The aim of this study was to evaluate the presence of gender differences among adult subjects affected by hyperacusis.

## 2. Materials and Methods

A literature search was conducted, focused on adult patients with hyperacusis, using the MedLine bibliographic database, to find relevant peer-reviewed studies published in the last 20 years. The keyword “Hyperacusis” [Mesh] was used to select the studies of interest. Additional filters were the publication year (2002 to 2022) and the age of the subject (>18 years).

Inclusion criteria were:

- Studies on the adult population.
- Studies that provide gender information of the participants.
- Studies including a pure tone audiometry assessment.

Exclusion criteria were:

- Studies published in languages other than English.
- Studies on pediatric populations.
- Studies not including pure tone audiometry.
- Studies published before 2002.

**Figure 1:**
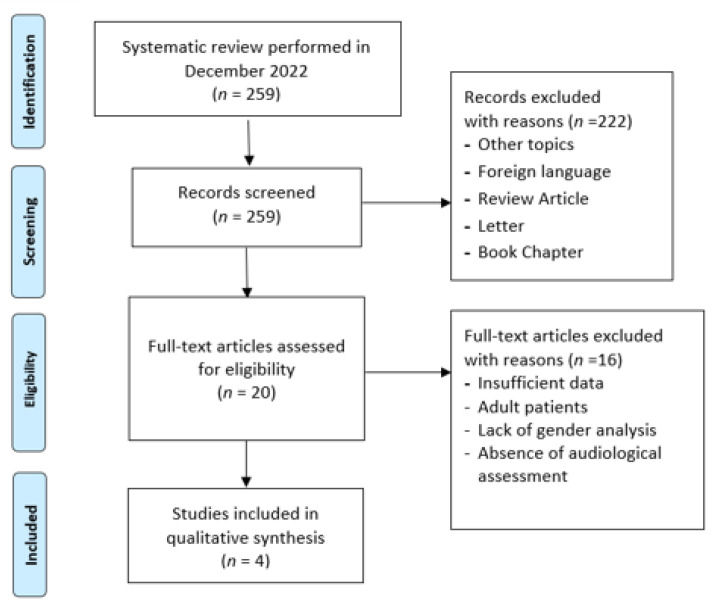
Flow diagram of the literature search, according to the PRISMA criteria (http://www.prisma-statement.org/, accessed on 1 April 2023), with the various steps in the manuscript selection process. The initially identified 259 manuscripts were reduced to 4 after the application of the selection criteria.

The review was performed according to the PRISMA guidelines (Figure 1).

## 3. Results

The four candidate papers for this review, included data from 604 patients of which 282 resulted as affected by hyperacusis. All the selected studies were prospective, except one. The year of publication ranged from 2003 to late 2013. The mean age ranged from 52 to 57 years old. Three studies did not include any standard deviation of the assessed sample. The reported male to female patient ratio was 1.2:1 (see the data summary in Table 1).

Guimaraes et al. [10] selected 309 people with tinnitus (169 females and 140 males) and in this sample, 57 subjects were affected by hyperacusis. From those, 26 were females and 31 were males. They all underwent VAS for tinnitus: in this scale, 0 corresponds to no perceived tinnitus and 10 is the loudest sound someone can imagine. The median degree of tinnitus was 7 (see the analytic data in Table 2). Hyperacusis was reported more frequently by male subjects (22.4%) than by female (15.3%), but the authors reported that there was no statistically significant difference between genders (*p* = 0.12).

In the study by Fioretti et al. [11], 37 subjects (19 females and 18 males) were evaluated by the Tinnitus Handicap Inventory test. The THI is composed of 25 items, each one with three possible answers: no (0 point), sometimes (2 points) and yes (4 points). The total score ranges from 0 to 100. Scores from 0 to 16 suggest no handicap; scores from 18 to 36 indicate a mild handicap; scores from 38 to 56 indicate a moderate handicap; and scores from 58 to 100 indicate a severe handicap. The information obtained by THI was implemented by the authors using a standardized questionnaire named Tinnitus sample case history (TSCH), based on the patient’s age, gender, family history of tinnitus, and tinnitus history. A total of 20 subjects were reported as affected by hyperacusis (10 females and 10 males). No gender differences in the outcomes were identified. Authors reported no statistically significant differences between genders according to THI scores.

Dauman R. et al. [6] described a total of 249 subjects (105 females and 144 males) assessed by the MASH test, an interview-based questionnaire that considers the level of annoyance in relation to the hypersensitivity to sound, based on a scale from 0 to 10. The results suggested that 197 subjects were affected by hyperacusis (81 females and 116 males), and that the prevalence of hyperacusis was similar in both genders. Interestingly they also reported that the perception of hyperacusis improves over time more than that of tinnitus.

Magalhaes et al. [12], assessed nine subjects (eight females and one male) and by administering a questionnaire on hyperacusis found that all eight female patients were affected. The small number of subjects in the study does not allow consideration of gender differences in the reported hyperacusis symptoms. They observed that female subjects are affected by hyperacusis more often than men, without a statistical difference and that tinnitus usually had preceded hyperacusis as a complaint in 78% of the subjects.

All patients included in this review, presented hyperacusis alongside tinnitus without a clear significance regarding the causative correlation and timing of occurrence, even if complaints about the tinnitus annoyance usually preceded those of hyperacusis.

Furthermore, a significant correlation between the level of annoyance due to tinnitus and hyperacusis cannot be established, nor between tinnitus and patients’ age and gender, although the discomfort of tinnitus was generally perceived as equal to or worse than that of hyperacusis. 

The MASH score and the overall annoyance showed a good correlation. Tinnitus annoyance was assessed as higher in females and severe in younger patients. Over time, the perception of hyperacusis, rather than that of tinnitus, has been reported to improve [4].

A significant correlation between sleep disorders and hyperacusis/tinnitus annoyance was also reported [9]. In this context, both the TSCH and THI are worthwhile screening tools to highlight sleep disorders and hyperacusis in patients presenting tinnitus as their main annoyance feature.

## 4. Discussion

Hyperacusis is a condition or symptom that can negatively affect the patient’s quality of life, being perceived as overwhelmingly loud or painful. Patients affected by hyperacusis often complain about specific situations or about sounds that they experience, that can increase their discomfort. In the audiological practice, tools specifically designed for assessing the hyperacusis etiology and annoyance are still missing, thus there is a limited comprehension of this symptom [19].

According to the literature, hyperacusis usually presents numerous other clinical manifestations, of which the main and most life-disabling is tinnitus [20]. Those complaining of these symptoms have reported a history of noise exposure, ototoxic drugs, or ear surgery as well. In fact, it has been already reported that noise and/or ototoxic drugs exposure can induce damages directly to the cochlea and the vestibule, but also to the central auditory system because of a reduced neural activity transmitted from the inner ear. According to data in the literature, tinnitus and hyperacusis could be the consequence of a tonotopic reorganization of the auditory pathway due to peripheral damage. An association between severe tinnitus and hyperacusis has been demonstrated in cross-sectional studies [21,22]. Several neurophysiological models of tinnitus and hyperacusis have been proposed, as this manifestation is possibly related to an increased state of cortical response (enhanced central gain) to sound and may be the explanation of the increase in the perception of loudness and thus the occurrence in those affected by hyperacusis [23,24,25]. Even if the description of this phenomenon in the literature is not very recent, it is still difficult to assess specific ear mechanisms by conventional electrophysiological testing, as there are no effective hyperacusis animal models [17,26].

Both, hyperacusis and tinnitus, could lead patients to seek the attention of a general practitioner or of a specialist, who should assess, investigate, and provide appropriate counselling.

A comprehensive audiological examination should be always performed and should include pure tone audiometry (0.125 to 16 kHz), the evaluation of uncomfortable loudness levels and tinnitus masking frequency [27]. When presenting with hearing loss, hyperacusis is often associated with tinnitus [8]. Specific self-assessment questionnaires have been developed for the evaluation of hyperacusis (and tinnitus), such as the MASH, THI, TSCH, t VAS, and should be always administered and evaluated.

Gender medicine, which is often underestimated, focuses on the differences in prevention, clinical symptoms, treatments, results, and social repercussions of the diseases among genders. Looking into the recent literature, gender and hyperacusis have been rarely addressed in the last twenty years [2,28]. Differences among genders in the auditory system have already been reported. Female subjects appear to have better hearing sensitivity, although male subjects have better skills at sound localization [29,30]. Furthermore, in addition to functional variations, objective auditory measures have been demonstrated to depend on gender, with females having larger amplitudes and shorter latencies of ABR waves I and V, compared to males.

Few studies reported how hearing features may differ among genders due to subcortical synaptic and axonal variations [31]; according to this hypothesis, hormones (i.e., estrogens) could influence the synaptic function of the auditory pathway [32,33].

Changes among genders may be related to cochlear and subcortical synaptic and axonal variations [31]. The menstrual cycle, but also the use of oral contraceptives have been reported to influence the function of the whole auditory system [18]. Since the cochlea contains estrogen and progesterone receptors, these hormones may impact their homeostasis over the menstrual cycle and hence modulate hearing functions indirectly. In support of this hypothesis, variations in electrode impedance have been recorded in women with cochlear implants during the luteal phase of the menstrual cycle [34]. Furthermore, other conditions related to gender, such as osteoporosis and osteopenia menopause, have been reported as possible risk factors for hearing loss and for alteration of vestibular function [35].

Despite not being widely investigated, the gender-related variances and subsequent biological differences might influence the development of hyperacusis as well as the symptoms correlated, such as tinnitus, hearing loss, anxiety and depression. None of the self-assessment questionnaires available address the gender issue, especially no specific information on sex differences and influences such as period, gestation or hormonal therapy are included in these tools up to date [4,5,6,7].

Gender differences have been described in audio-vestibular disorders in current years. It has been reported that the prevalence of presbycusis and of sudden hearing loss is higher among male than female subjects, while the prevalence of autoimmune inner ear disease and tinnitus, a symptom sometimes associated with hyperacusis, seem to be more prevalent in females [36]. To date, according to the data of this review, it is still not possible to identify any gender differences in hyperacusis patients. However, evaluating the data of the selected studies, it seems that hyperacusis can improve over time, more than tinnitus, and male subjects could show a higher annoyance towards hyperacusis. In addition, while tinnitus annoyance has been described as higher in females and more severe in younger patients, the perception of hyperacusis over time have been reported to improve, especially in women [4].

In the literature, several risk factors have been linked to tinnitus, such as depression, anxiety, exposure to recreational noise, smoking; none of these factors seems to be related to hyperacusis, so far [20,21,22].

Currently, a definitive treatment for hyperacusis, medical or surgical is still not available; its management options can consist in different strategies that can help reduce the discomfort of sensitivity to sounds. These include drugs, but also the prescription of hearing aids (when hearing loss is also present), noise generators and cognitive behavioral therapy (CBT). In patients fitted with hearing aids, the persistence of hyperacusis over time has been observed, even if with a reduced discomfort. Regarding noise generators, considering different fitting times, the MASH score of those using noise generators was comparable to that of the whole population. In particular, it has been reported that when hyperacusis is associated with hearing loss, it has been reported that a transient noise reduction feature, allowing the attenuation of loud and impulsive sounds, can represent a benefit in the fitting of hearing aids. In other cases, the use of continuous low-level white or pink noise has also been reported to offer a benefit to hyperacusis patients, therefore using hearing aids also as a sound generator [37].

However, an improvement of the distress related to the hyperacusis perception was observed more frequently than tinnitus and can eventually occur spontaneously over time [37,38,39,40].

Because tinnitus and hyperacusis share various features, tinnitus retraining therapy and cognitive behavioral therapy have been used to treat both with comparable outcomes [41]. Especially CBT has been reported to have positive effects regarding tinnitus severity (TQ), hyperacusis and psychological strain (BSI and BDI-II) [23,42].

Further research is required and may be key in establishing tailored treatments, reducing the perception of discomfort, the general distress or somatic and psychosomatic symptoms [13]. In fact, according to the most recent findings on gender medicine, the anatomo-physiologic differences between sexes may contribute to the different manifestations of the diseases, including inner ear disorders, therefore also representing a possible key source for future tailored therapeutic approaches.

Limitations of the study: A first limitation is the lack of information about the causes or comorbidities in patients with hyperacusis. This information can serve to better define the etiology and therefore to solidify the eventual presence of gender differences. Furthermore, a specific assessment of the relationship between hyperacusis and tinnitus, which often co-exist, is desirable. A secondary limitation is the fact that in the hyperacusis data factors influencing the gender and its severity are missing. Hyperacusis severity seems to be related more to the age factor (i.e., more severe in the youngest patients) than to gender. In addition, information about familial history of hearing loss, tinnitus and hyperacusis is lacking in the current literature; if available, this could have added some clues about the eventual heritability of this disorder. Therefore, in order to bridge this information gap, specific questionnaires should be set up to assess the hyperacusis discomfort [43,44].

## 5. Conclusions

Hyperacusis is a symptom that can severely affect patients’ quality of life, and a comprehensive understanding of its features is necessary. Furthermore, the comprehension of the role that tinnitus could play as a predisposing factor for hyperacusis onset is also significant.

According to the results of this review, males and females exhibit a similar level of hyperacusis. However, in light of the subjective nature this symptom, the eventual set up of further tests for the evaluation of hyperacusis features and significance could be very helpful. Furthermore, additional studies are also necessary in order to evaluate the long-term effects and consequences of hyperacusis on gender.

## Figures and Tables

**Table 1:** Epidemiological features of hyperacusis within the selected studies. Under the Study column, the symbol “R” corresponds to Retrospective study, while the symbol “P” to Prospective study. In the last column, the symbols F and M next to the patient numbers correspond to “Female” and “Male”. %♀ = percentage of females affected by hyperacusis, %♂ = percentage of females affected by hyperacusis.

n	Author	Year	Country	Study	Mean Age	Total Cohort	Gender	Affected	%♀	%♂	Pure Tone Audiometry (Outcomes)
1	Guimarães AC et al. [16]	2013	BRAZIL	R	53	309	169 F; 140 M	57 (26 F; 31 M)	15.3%	22.4	Performed but not described
2	Fioretti AB et al. [17]	2013	ITALY	P	57.2+/−14.1	37	19 F; 18 M	20 (10 F; 10 M)	52.6%	55.5	2 normal HL9 SNHL
3	Dauman R et al. [6]	2005	FRANCE	P	52/54	249	105 F; 144 M	197 (81 F; 116 M)	77.1%	80.5	The hearing loss did not correlate directly with the level of hyperacusis
4	de Magalhães SL et al. [18]	2003	BRAZIL	P	55,32	9	8 F; 1 M	8 (8 F; 0 M)	100%	0	Normal HL
		2003–2013				604	301 F; 303 M	282 (125 ♀; 157 ♀)	41.5%	51.8	

**Table 2:** Hyperacusis assessment within the selected studies. VAS = Visual Analogue Scale; MASH = Multiple-Activity Scale for Hyperacusis; THI = Tinnitus Handicap Inventory Test; TSCH = Tinnitus sample case history; PTA = Pure tone audiometry; OAE = Otoacoustic emission; SAT = Speech audiometry test; IT = Immittance test.

TEST	Guimarães et al. [16]	Fioretti et al. [17]	Dauman et al. [6]	Magalhães et al. [18]
Questionnaire	VAS	(a) TSCH (b) THI	(a) MASH; (b) Structured interview (overall annoyance of hyperacusis and hearing deficiency, scale from 0 to 10); (c) Iowa Tinnitus Handicap Questionnaire	-
Audiological evaluation	PTA SAT IT	PTA IT OAE.	PTA SAT	PTA SAT OAE
Clinical assessment Other	Neurologic; ENT-	ENT-	--	ENT Laboratory tests
Hyperacusis Grading	VAS from 1 to 10: Hyperacusis present in 57 (18.4%) patients at an intensity ranging from 1 to 10 and a median of 5.	TSCH 20 patients (54%) reported hyperacusis (grading not provided).	5 groups based on annoyance score (a) no hyperacusis; (b) mild; (c) moderate; (d) substantial hyperacusis; (e) severe hyperacusis.	(a) 4 moderate; (b) 1 severe; (c) 1 mild
Results	The presence of hyperacusis more frequent in the male gender; 31 (22.4%) men and 26 (15.3%) women.There was no statistically significant difference between genders (*p* = 0.12).	No statistically significant differences between genders according to THI scores TSCH (a) 20 patients reported sleep disorders (54%); (b) 20 patients reported hyperacusis (54%); (c) 11 patients (30%) sleep disorders + hyperacusis. THI (d) slight (1 pt,16%), (e) mild (6 pt, 32%), (f) moderate (7 pt, 30%), (g) severe (5 pt, 19%), (h) catastrophic (1 pt, 3%).	Comparable prevalence in both genders: males (80.5%; 116/144); females (77.1%; 81/105). Improvement over time is better for Hyperacusis (19/30; 63.3%), than for tinnitus (15/32); 46.8%).	Women are affected more often than men. Tinnitus preceded hyperacusis as a complaint in 78% of the subjects. No direct correlation between the severity of tinnitus and of hyperacusis

## Data Availability

Not applicable.
